# Novel OCT findings in choroidal osteoma: brief report

**DOI:** 10.1186/s40942-021-00317-5

**Published:** 2021-08-17

**Authors:** Ricardo Luz Leitão Guerra, Rafael Cicconi Arantes, Eduardo Ferrari Marback, Carol L Shields

**Affiliations:** 1 Retina Department, Leitão Guerra - Oftalmologia, Rua Rio de São Pedro, 256, Bahia 40150-350 Salvador, Brazil; 2 Ophthalmology Department, Faculdade de Medicina da Bahia, Salvador, Brazil; 3 Ocular Oncology Department, Wills Eye Hospital, Philadelhia, United States

**Keywords:** Choroidal osteoma, OCT, Optical coherence tomography, Ocular pathology, Choroidal cavern, Choroidal loculation

## Abstract

The aim of this article is to report the presence of choroidal loculation of fluid and choroidal cavern in a case of choroidal osteoma, previously undescribed in this disease.

Dear editor,

Choroidal osteoma is a rare intraocular tumor that can simulate several intraocular conditions. The clinical appearance on fundus examination, aided by ultrasound, are tools for an accurate diagnosis [1].

The evolution of optical coherence tomography (OCT) techniques for choroidal evaluation, such as enhanced depth imaging (EDI), have allowed detailed analysis of choroidal tumors [2].

Intrinsic and unique features of choroidal osteoma hmacular neovascularizationave been described using EDI-OCT such as “subtle horizontal hyperreflective lamellar lines (bone lamella) with occasional denser lines (cement lines), speckled (spongy, cancellous) tissue, and horizontally or vertically oriented tubular channels representing Haversian or Volkman canals or cavernous vascular spaces” [2].

Other researches have also described retinal pigmented epithelium (RPE) and external retinal layer abnormalities in the deossified portion of the tumor [3], subretinal fluid [4], macular neovascularization (MNV) [4], irregular tumor surface [3], focal choroidal excavation [4] and osteoma protrusion into the vitreous [4] as OCT findings in choroidal osteoma.

Reviewing the OCT b-scans of a 30-year-old male patient with a large macular choroidal osteoma, we noted two additional OCT findings, previously unreported, including “choroidal loculation of fluid” and RPE tear over “choroidal cavern” (possibly deossified choroidal osteoma) (Fig. 1).

The term “choroidal loculation of fluid” has been used to describe hyporeflective spaces located in the outer choroid with an angular inner border and larger size than the largest choroidal vessels [5]. This finding has been observed in central serous chorioretinopathy (CSC) and has been suggested that it might serve to drive fluid into the sub-RPE and subretinal spaces [5]..

The presence of choroidal loculation of fluid in the present case might be due to the lack of permeability of the choroidal bone tissue. However, the exact mechanism in a single case report might not be representative. We believe that, as hypothesized for CSC, choroidal loculation of fluid can play a role in the presence of subretinal fluid in choroidal osteoma patients without MNV.

“Choroidal caverns” spectrum of lesions is a recent proposed classification of hyporeflective areas within the choroid based on similar morphology and includes intrachoroidal cavitation, choroidal cleft, and choroidal lipid globule caverns [6].

“Speckled hyperreflective dots” found in choroidal osteoma on OCT are suggestive of small trabecular bone tissue [2] and, areas of hyporeflective spaces without hyperreflective dots have been suggestive of areas of presumed osteoclastic activity [1]. In the present case, we observed an overlying RPE tear and cavern and speculate on their origin.

In summary, we would like to highlight these new findings to encourage clinicians to investigate the imaging features and potential pathophysiology of choroidal osteoma.

**Fig. 1:**
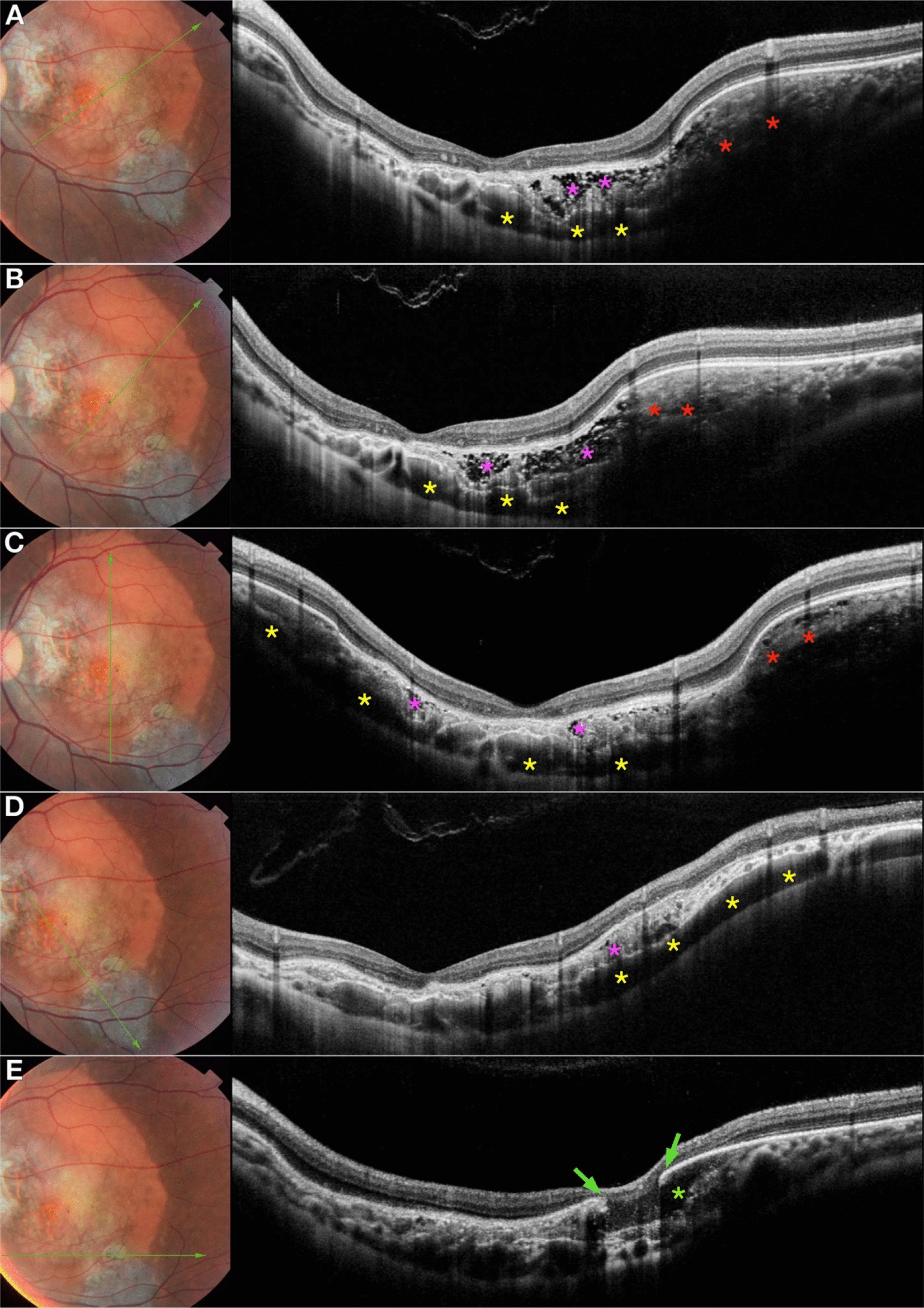
Enhanced deep imaging optical coherence tomography of a choroidal osteoma. **A**, **B** and **C** Sponge-like appearance in the desossified area (purple asterisk). Lamellar appearance and posterior shadow at the ossified area (red asterisk). Hyporeflective spaces located in the outer choroid corresponding to choroidal loculation (yellow asterisk). **D** Sponge-like appearance in the desossified area (purple asterisk). Hyporeflective spaces located in the outer choroid corresponding to choroidal loculation (yellow asterisk). **E** Edges of the retinal pigmented epithelium tear (green arrow) overchoroidal cavern (green asterisk)

## Data Availability

The datasets used and/or analysed during the current study are available from the corresponding author on reasonable request.

## Abbreviations

OCT: Optical coherence tomography
EDI: Enhanced depth image
CSC: Central serous corioretinopathy
MNV: Macular neovascularization
RPE: Retinal pigmented epithelium
